# Stable resynthesized *Brassica napus* lines show similar meiotic behaviour to established *B. napus*

**DOI:** 10.1007/s10577-026-09799-1

**Published:** 2026-04-07

**Authors:** Vinita Ramtekey, Elizabeth Ihien Katche, Mariana Baez, Zhenling Lv, Annaliese S. Mason

**Affiliations:** 1 Plant Breeding Department, Institute for Crop Science and Resource Conservation, University of Bonn, Kirschallee 1, 53115 Bonn, Germany; 2 ICAR-Indian Institute of Seed Science, 275103 Mau, India

**Keywords:** Meiotic stability, Cytogenetics, Chromosome pairing behaviour, Rapeseed, Oligo-FISH

## Abstract

*Brassica napus* (rapeseed/canola) is an allotetraploid (AACC, 2*n* = 4*x* = 38) resulting from spontaneous hybridization between *B. rapa* (AA, 2*n* = 2*x* = 20) and *B. oleracea* (CC, 2*n* = 2*x* = 18). Although established *B. napus* is meiotically stable, resynthesized lines (2*n* = AACC) produced by hybridizing between progenitor species *B. rapa* and *B. oleracea* are usually meiotically unstable, and show frequent chromosomal rearrangements caused by homoeologous recombination between the A and C genomes. Previously, we identified resynthesized rapeseed lines showing contrasting levels of homoeologous recombination, as assessed by genotyping for copy number variants. Here, we aimed to characterise meiotic chromosome pairing behaviour in fifteen resynthesized lines representing putatively stable, unstable and intermediate types. Putatively stable lines showed predominantly normal meiosis (average 91% bivalent formation), while putatively unstable lines showed frequent abnormalities such as multivalent formation (average 60% bivalent formation). Univalents were unexpectedly rare in Metaphase I. A1-C1 specific probes revealed that stable lines showed approximately 18% A-C pairing (7/40 pollen mother cells), not significantly different to the 13% A-C pairing (5/40 pollen mother cells) in established *B. napus*, but in contrast to the unstable line with 46% A-C pairing (25/54 pollen mother cells). Surprisingly, all resynthesized lines initially classed as having intermediate stability based on copy number variation showed either stable or unstable-type meiotic behaviour, suggesting a more qualitative rather than quantitative phenotype. Our results suggest that differences in multivalent formation frequencies and homoeologous A-C pairing differentiate stable and unstable lines, confirm the production of meiotically stable synthetic *B. napus*, and provide a basis for further investigation of genetic factors contributing to this effect.

**Supplementary Information:**

The online version contains supplementary material available at 10.1007/s10577-026-09799-1.

## Introduction

Polyploidization is a major driving force in evolution and speciation (Leitch and Leitch 2008), and is particularly common in angiosperms (Jiao et al. 2011). Polyploids often exhibit genome buffering, enhanced heterozygosity, and novel phenotypic and genotypic variation compared to their diploid counterparts, which can be attributed to various interconnected mechanisms such as genome rearrangements, altered gene dosage, gene expression and regulation and epigenetic modification (Pelé et al. 2018; Doyle & Coate 2019; Heslop-Harrison et al. 2023). Polyploids are usually categorised as either autopolyploid (sets of chromosomes originating from the same species), or allopolyploid (two or more genomes derived from different species) (Kihara and Ono 1926).

Allopolyploids which arise from hybridization between two different sets of chromosomes (genomes) face the major challenge of differentiating between ancestrally related (homoeologous) chromosome copies (Pelé et al. 2018). During the course of evolution, the major adaptation that has been observed in established allopolyploids is their ability to distinguish between homoeologous and homologous chromosomes when choosing meiotic recombination partners. Prevention of extensive pairing between non-homologous (homoeologous or otherwise) chromosomes is critical for ensuring regular segregation of chromosomes into subsequent gametes without putative issues such as loss of chromosomes or chromosome segments, which is potentially detrimental to plant fertility and viability (Mercier et al. 2015). However, how established allopolyploids stabilise meiosis is still unknown in most taxa (Bomblies 2023).

There are several ways in which allopolyploids might stabilise meiosis. Crossovers between homoeologous chromosomes could be prevented completely, resolved in such a way that no recombination can occur between homoeologues, or simply made extremely rare due to strong preference for homologous over homoeologous crossovers, even in the absence of specific genetic factors that suppress pairing between homoeologous chromosomes (Bomblies 2023). In allohexaploid bread wheat, which is one of the most extensively studied species with respect to understanding of the establishment of meiotic stability in allopolyploids, the *Ph1* gene acts not only to suppress homoeologous chromosome recombination but also to promote homologous recombination (Riley & Chapman 1958; Griffiths et al. 2006; Bhullar et al. 2014). Other genetic factors acting to prevent homoeologous recombination have been identified in *Arabidopsis suecica* (Henry et al. 2014) and *Brassica napus* (Jenczewski et al. 2003; Liu et al. 2006; Higgins et al. 2021), although the mechanism of action of these genetic factors is so far unknown. Jenczewski et al. 2003 identified a genetic factor *PrBn* which influences homoeologous crossover frequency in *B. napus* haploids, and Nicolas et al. 2009 observed differences in homologous recombination frequency in allotriploid *Brassica* hybrids (AAC) produced with different *PrBn* types, suggesting *PrBn* could have dosage sensitive effects on recombination. However, the gene corresponding to this locus has not yet been identified or fully functionally characterized. Recently, Gonzalo et al., (2019) demonstrated that reduced expression of *MSH4*, belonging to the ZMM-group of class I crossover pathway proteins, significantly decreases the occurrence of homoeologous recombination, while having minimal impact on homologous recombination in *B. napus*. However, the specific impact on partner choice in the context of pairing or recombination regulation in *Brassica* is still uncertain. As suggested by earlier QTL mapping results (Jenczewski et al. 2003; Liu et al. 2006), an integrated system of multiple genes is most likely associated with meiotic stabilization in *B. napus*, such that the molecular basis of meiotic stability involves polygenic adaptation to allopolyploidy.

Neo-allopolyploids and resynthesized hybrids are a useful model with which to investigate mechanisms underlying meiotic stability. In most newly synthesised allopolyploids (produced by crosses between lower ploidy parents), meiosis is associated with numerous abnormalities, including but not limited to incorrect synapsis, homoeologous recombination, chromosome bridges, and chromosome mis-segregation (anaphase I). These meiotic abnormalities can lead to aneuploidy, chromosome rearrangements and deletions and duplications of chromosome segments, which may result in loss of fertility and viability in subsequent generations (Bomblies 2023; Pelé et al. 2018; Xiong et al. 2021). Studies of synthetic hybrids have previously investigated cytological causes of meiotic stability. Madlung et al. (2005) observed about 30% meiotic abnormalities in the form of chromosome breakage, bridges and rearrangements in synthetic *Arabidopsis* allopolyploids, indicating increased meiotic instability compared to their parents (10%). Similarly, Chéron et al. 2024 suggested that associations between incorrect recombination partners and homoeologous recombination contribute to meiotic instability in neo-synthetic allopolyploid *A. suecica*.

Recently, synthetic *Brassica napus* has emerged as an important model system to study meiosis in allopolyploids (Katche & S. Mason 2023; Bomblies 2023). Allotetraploid *B. napus* (AACC, 2*n* = 4*x* = 38) is the product of natural interspecific hybridization coupled with polyploidization between diploid ancestors of *Brassica rapa* (AA, 2*n* = 2*x* = 20) and *Brassica oleracea* (CC, 2*n* = 2*x* = 18) around 7500 years ago (U, 1935; Chalhoub et al. 2014). Established *B. napus* is a relatively meiotically and genomically stable allopolyploid which shows diploid-like meiosis, including predominantly homologous recombination even in the presence of homoeologous chromosomes (Jenczewski et al. 2003). By contrast, synthetic *B. napus* (formed by either *B. rapa* × *B. oleracea* or *B. oleracea* × *B. rapa*) is usually meiotically unstable, but the cause is still unknown (reviewed by Katche & Mason 2023). Cytogenetic, molecular and genome sequencing studies have revealed that resynthesized *B. napus* often display genetic changes as well as homoeologous rearrangements (Gaeta et al. 2007; Xiong et al. 2011, 2021; Chalhoub et al. 2014; Katche, et al. 2023a, 2023b; Davis et al. 2023), leading to genomic copy number variants (deletions, duplications, and translocation) as well as presence/absence variation (Katche et al. 2023a; Schiessl et al. 2019). Such variants are most common in chromosomes which are structurally conserved (syntenic along the whole length of the chromosome) between subgenomes, such as A1 and C1, and A2 and C2 (Xiong et al. 2021; Higgins et al. 2021). Unlike resynthesized *B. napus*, established cultivars show rarer or lower rates of homoeologous rearrangements (Parkin et al. 1995; Sharpe et al. 1995; Howell et al. 2008; Udall et al. 2005). In the past decades many cytogenetic studies have extensively confirmed with the help of fluorescently labelled probes as well BAC-FISH that homoeologous recombination between the closely related A and C subgenomes is a major feature of resynthesized *B. napus* (Howell et al. 2008; Xiong et al. 2021; Xiong & Chris Pires 2011).

In our previous work, we characterised a large set of resynthesized rapeseed material which was previously developed via hand pollination followed by embryo rescue between *B. rapa* and *B. oleracea* cultivars (domesticated winter-type accessions from different cultivated subspecies) followed by colchicine treatment then self-pollination for one or more generations (Rygulla et al. 2007; Girke et al. 2012, and Jesske et al. 2013). Within this material we identified several later-generation synthetic *B. napus* lines from different genetic backgrounds which accumulate no or very few new copy number variants after self-pollination and are therefore putatively stable (Katche et al. 2023a), unlike all previously produced resynthesized *B. napus* lines (Katche et al. 2023b). In the present study, we aimed to investigate and characterise meiosis in these lines and compare between these putatively “stable” and “unstable” resynthesized *B. napus* types as well as established *B. napus*. We hypothesised that meiosis in the putatively stable lines would be normal and similar to meiosis as established *B. napus*, in contrast to unstable resynthesized *B. napus*, and aimed to characterise meiotic progression in order to better elucidate the cytological mechanisms responsible for these differences.

## Material and methods

### Plant material

Production of the resynthesized winter *B. napus* allotetraploid (AACC) lines used in the present research is described in Rygulla et al., (2007), Girke et al., (2012), and Jesske et al., (2013). The plant material consists of domesticated winter-type resynthesized *B. napus* derived from hybridization followed by embryo rescue between different cultivated subspecies of vegetable-type *B. rapa* (AA) and *B. oleracea* (CC) followed by one or more round of self-pollination. Later Katche et al., (2023a) characterized these lines as putatively stable or unstable based on frequencies of novel and inherited CNVs in each line.

For the first year of flower bud collection, plants were grown at Justus Liebig University Giessen, Germany, as described by Katche et al., (2023a). In the subsequent generation, only putatively stable and unstable resynthesized lines with a minimum of three seeds per line were germinated in quick-pots during the year of 2024. Germinated plants at the 4–6 leaf stage were vernalized at 4—6°C for minimum 14–16 weeks (December 2023 to March 2024) in a controlled environment room at Campus Klein Altendorf, the University of Bonn field station. After vernalization, plants were transferred to the glasshouse and grown in 10 L pots under heated glasshouse conditions (minimum 20 °C day and 16 °C night, but up to 35 °C on hot days in late summer) at the University of Bonn, Poppelsdorf from April to September 2024.

In the present study we selected fifteen resynthesized *B. napus* lines from Katche et al., (2023a) and categorised these into three groups based on CNV data: 1) no novel CNVs = “stable”, 2) 1–3 novel CNVs = “intermediate”, and 3) > 8 CNVs = “unstable” (Table S1). For the putatively stable lines (with the exception of R76), the maternal parent was *B. oleracea* and the paternal parent was *B. rapa,* with different subspecies for both parents (Table S1 and Fig. S1). For the putatively unstable lines (with the exception of OLY21), the maternal parent was *B. rapa*, while paternal parent was *B. oleracea* (Table S1 and Fig. S1). The putatively highly stable lines were > S3 generation, while the putatively highly unstable were either S1 or S2 generation (Fig. S1; Katche et al. 2023a). As a reference for stable meiosis, we used *B. napus* cv. Drakkar (received from INRAE, France). Additionally, we selected one putatively stable (L16) and unstable (OLY21) resynthesized line to investigate meiotic progression and A1/C1 pairing, as these lines did not have any apparent fixed translocations involving chromosomes A1 and C1 (Katche et al. 2023a).

### Seed fertility in putatively stable and unstable resynthesized lines

Three individual plants per genotype were bagged using microperforated plastic bags to encourage self-pollinated seed set. The average number of total self-pollinated seed set was measured for each of the three plants per genotype to assess seed fertility differences between putatively stable and unstable resynthesized lines.

### Inflorescence fixation, slide preparation and meiotic observation

Immature unopened flower buds were collected in Carnoy’s I fixative solution (absolute ethanol: glacial acetic acid 3:1, v/v) for 24—48 h at room temperature to fix the cells at the respective meiotic stages (Windham et al. 2020). Later, the fixed flower buds were transferred to 50% ethanol for further downstream analysis as well as long term storage. Out of six anthers, one anther was squashed and stained with 1% acetocarmine to identify meiotic stages while the remaining five anthers were used for slide preparation via enzymatic digestion (composition of 1% pectyolase Y-23 and 2% cellulase “Onozuka” R-10 in 0.1M citrate buffer)(Kirov et al. 2014). Slide preparation involved submerging the remaining five anthers in an enzymatic mixture of 20 µl in a 0.5 ml tube and incubating for 1—1.5 h at 37 °C followed by washes with 70% ethanol. Subsequently, anthers were scrambled with a needle in 60—80 µl of 70% ethanol to form a white musky coloration, which gives a pellet upon quick centrifugation (25–30 s) using a benchtop centrifuge (FastGene® Mini Centrifuge (NG002P)). The pellet contains meiocytes as well as some somatic tissue (anther wall and tapetum). The ethanol supernatant is removed and pellet dried. Once the pellet is dried, we add 100% GAA (approximately 25—40 µl depending on the pellet size) and vortex thoroughly. Finally, we use the dropping method to prepare slides containing meiocytes and incubate for another 5 min in a humid chamber at room temperature (RT) to evenly spread the meiocytes. A drop of Vectashield® antifade mounting medium containing 4’, 6- diamidino-2-phenylindole (DAPI) (H-1200–10, Vector Laboratories) was used on slides to stain and visualize the chromosomes inside meiocytes for cells undergoing meiosis). These slides were finally used to investigate chromosome pairing behaviour at diakinesis of prophase I of meiosis I from male meiocytes. In order to understand the overall meiosis progression of the putatively stable and unstable resynthesized lines, we focused on metaphase I, anaphase I, telophase I, metaphase II, and anaphase II/telophase II. Visualization of meiocytes was performed using an inverted fluorescence microscope (Zeiss Axio Imager M2) with a 40 × and 100 × oil immersion lens and up to 10 000 × total magnification. Images were captured using the Zeiss software ZEN Blue (version 3.2), which was also used for cropping, size adjustment and contrast optimization of microscopic images. A minimum of 30 cells per line was analysed for a robust interpretation of meiotic chromosome behaviour at diakinesis. Chromosome configurations were scored as bivalent, univalent or multivalent at diakinesis. Other chromosomal aberrations such as anaphase bridges, laggards or chromosome fragments at anaphase/telophase stages of meiosis I and meiosis II were also observed.

### Probe labelling, oligo-FISH and imaging

In order to clearly identify univalent, bivalent and multivalent chromosomes, DNA from centromere repeat-specific sequences CentBrI and CentBrII (Xiong and Chris Pires 2011) were labelled with Cy5-dUTP, using a nick translation kit (11745808910, Roche) as described by (Kato et al. 2006). Specific labelling of A1 and C1 was done using A1-specific oligo probes labelled with ATT0550 (red) and C1-specific oligo probes labelled with ATT0488 (green) (by Arbor Biosciences, via BioCat GmbH, Germany). FISH was performed according to Han et al., (2015) and Montenegro et al., (2022) with slight modification. We used all three DNA probes (CentBrI and CentBrII were combined to form one centromere probe and A1 and C1 were used as second and third DNA probes) at the same time on a single slide. Hybridization started with fixing the slides in Carnoy’s I solution for 30 min followed by dehydration with 100% ethanol for 15 min. Once the slides dried out, we treated with pepsin (50 µg/ml) for 30 min at 37 °C in a humid chamber followed by two successive washes with 2 × SSC buffer (saline sodium citrate buffer) at RT. Later we treated slides with 4% formaldehyde and incubated for 10 min at RT, followed by two successive washes with 2 × SSC buffer then dehydration with an ethanol series (70% and 100% ethanol). After these treatments, we air dried the slides for at least an hour and applied the hybridization mixture to the meiotic cell zone on the slides. The hybridization mixture comprised of 50% formaldehyde, 10% dextran sulphate, 2 × SSC buffer, 200 ng A1 and C1 oligo probes and 50 ng of centromere probes. Slides were incubated at 75 °C for 5–7 min after adding hybridization mixture to the slides and later hybridized for 2 days at 37 °C in a moisture chamber, in the dark. After a long incubation, we washed slides once with 2 × SSC buffer at RT and at 55 °C for about 20 min followed by another wash at RT with 2 × SSC buffer. Once the slides partially dried, the Vectashield® antifade mounting medium containing DAPI (H-1200–10, Vector Laboratories) was used on slides to capture the images using an inverted fluorescence microscope (Zeiss Axio Imager M2) with a 40 × and 100 × oil immersion lens with different filters and software described above. A minimum of 30 microscopic images/line were analysed. Univalent, bivalent or multivalent behaviour of chromosomes A1/C1 was scored at diakinesis.

### Statistical data analysis

Statistical analysis was performed in R studio v 4.4.1 (R Core Team (2024) https://stat.ethz.ch/pipermail/r-announce/2024/000704.html). Visualization plots for seed fertility and meiotic chromosome pairing behaviour (bivalent, univalent and multivalent) of putatively stable and unstable lines were produced using the “ggplot2” package available in R (Wickham 2016). A Shapiro–Wilk normality test was carried out to assess normality of seed fertility and meiosis confirmation data. Seed set was normally distributed, so we performed ANOVA followed by Tukey’s HSD test to check for significant differences between putatively stable and unstable resynthesized lines. Meiotic configuration data was not normally distributed, so a non-parametric Kruskal–Wallis test was used to check for significant differences between groups and between genotypes followed by a pairwise Dunn’s post hoc test for significant comparisons with multiple testing correction using the Bonferroni method. The adjusted p-values were used to cluster groups and genotypes into sets by significant differences between them, which were indicated by different letters in the plot. Fisher’s exact test for count data was used to detect the significance for A1/C1 homoeologous pairing between putatively stable and unstable resynthesized lines. The Pearson correlation coefficients were computed by using ‘corrplot’ package available in R (Wei & Simko 2024).

## Results

### Putatively stable resynthesized lines exhibited higher seed set, in contrast to unstable lines

**Fig. 1:**
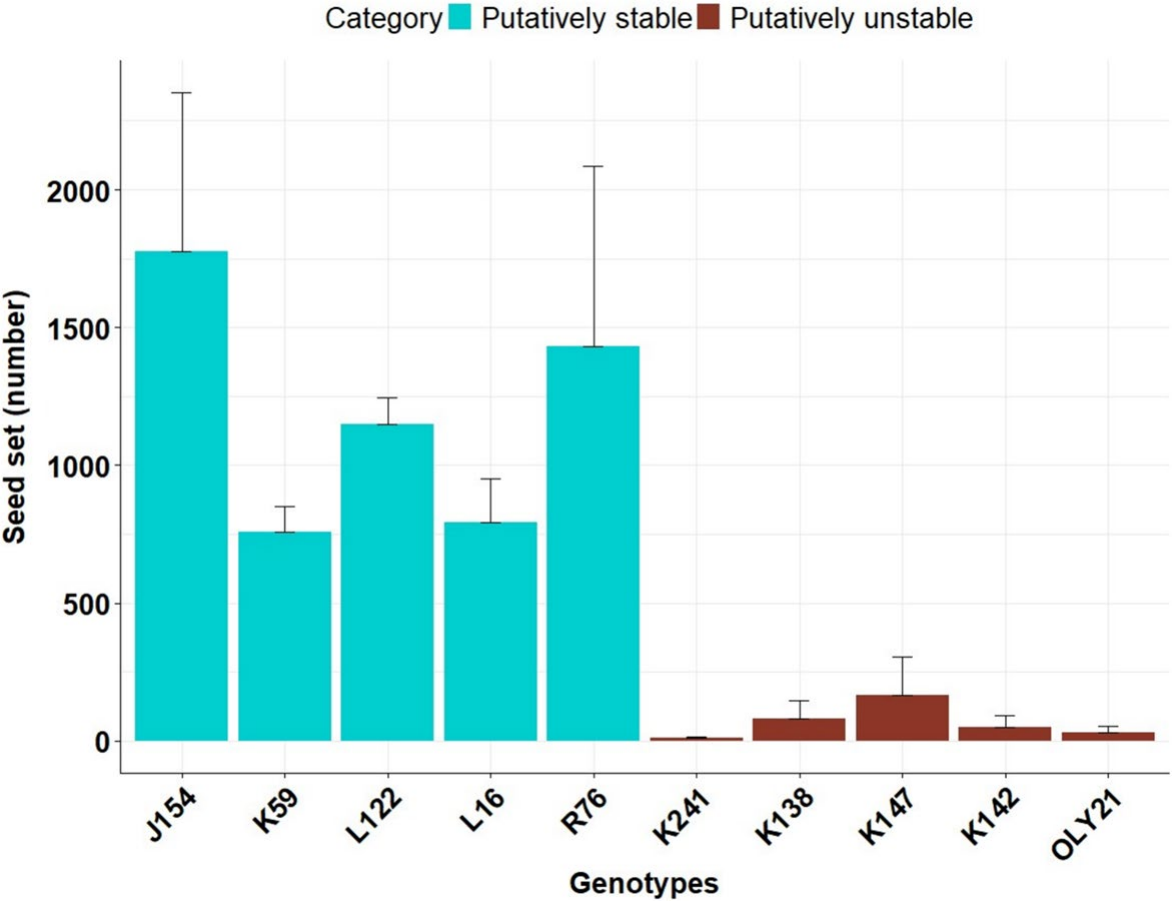
Seed fertility (total seeds per plant) in putatively stable and unstable resynthesized lines

Putatively stable lines showed higher seed set. Total self-pollinated seeds ranged from 499 to 2720 (on an average 1181) per plant across putatively stable resynthesized lines which was significantly higher (Fig. 1, Table S2, ANOVA, P = 0.01, followed by Tukey’s HSD P < 0.01) than seed set in unstable lines, which ranged from 0 to 444 seeds per plant (average 52) (Table S2).

### Putatively stable resynthesized *B. napus* lines exhibited similar chromosome pairing behaviour to established ***B. napus***, over two generations, in contrast to unstable lines

**Fig. 2:**
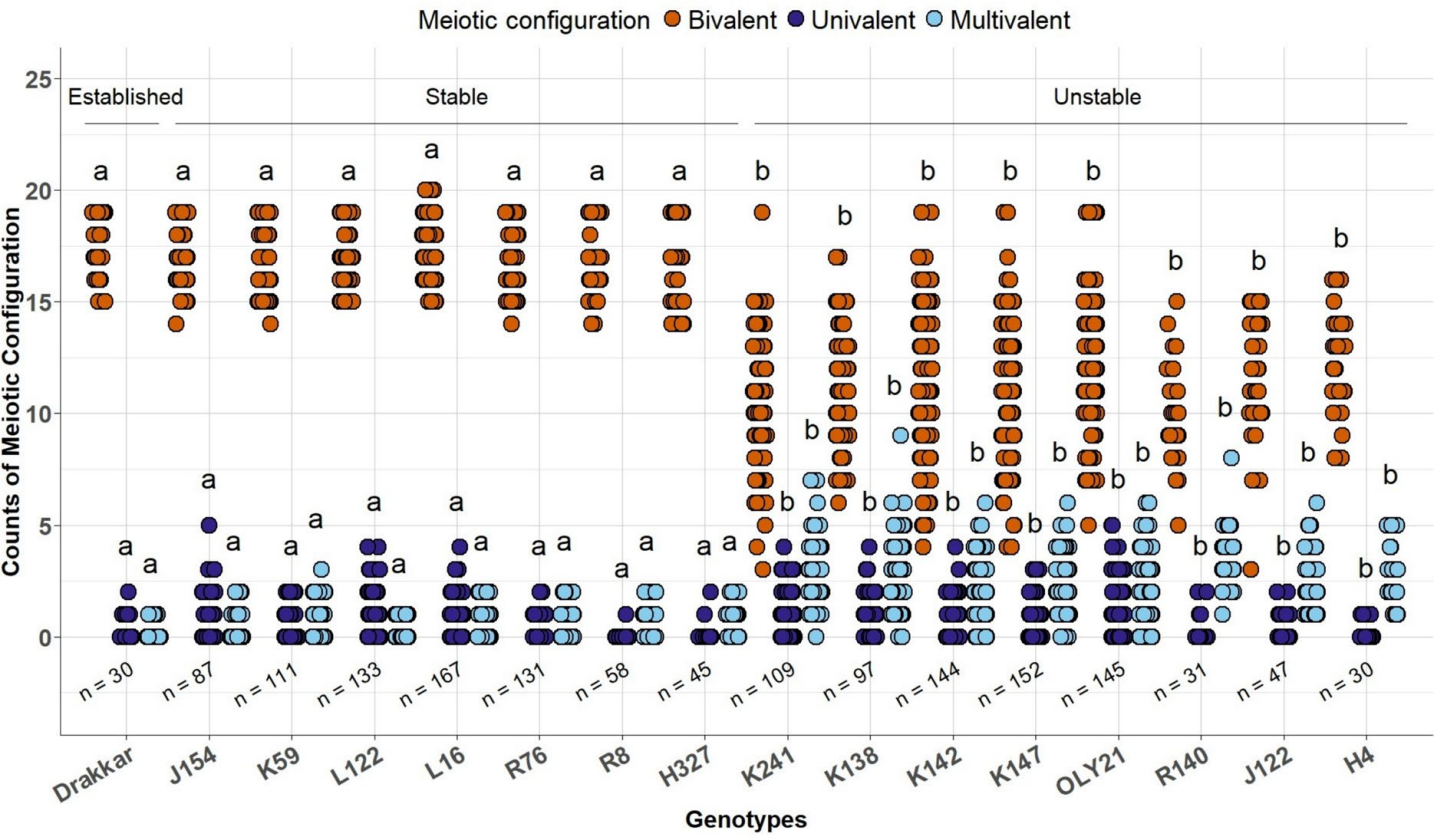
Meiotic chromosome pairing behaviour in established *Brassica napus* cultivar “Drakkar” and in putatively stable and unstable resynthesized *B. napus* lines. Letters indicate significant differences between lines for each of bivalent, univalent and multivalent frequencies (Kruskal–Wallis test followed by Dunn’s post hoc test, p < 0.05). n = number of meiotic cells. Each dot represents a single meiotic cell

Putatively stable lines displayed meiotic chromosome pairing behaviour which was statistically similar to that of established *B. napus* cultivar “Drakkar” in terms of univalent, bivalent and multivalent frequencies (p > 0.05, Fig. 2, Table S3). The putatively stable group showed higher frequencies of bivalents (91% on an average), than the putatively unstable lines (60% on average; Fig. 2, Table S3; p < 0.001). There was a significant difference between univalent frequencies in stable lines (2%, p < 0.001) compared to unstable lines (1%), as well as for multivalent formation (3% in established and stable, p < 0.0001) compared to unstable lines (13%) (Table S3; Fig. 2).

In the first experimental year and generation, putatively stable lines exhibited 1% univalent frequency and 3% multivalent frequency on average, while unstable lines averaged 1% univalent frequency and 12% multivalent frequency (Table S3). However, only the average multivalent frequencies showed statistically significant differences between putatively stable and established *B. napus* as well as between putatively stable and unstable resynthesized lines (p < 0.001 for stable and for established, Fig. S2). Similar results were observed in the subsequent generation in the resynthesized lines, where the subsequent generation was produced from self-pollinated seeds collected from the previous generation plants, except where univalent frequencies were also found to be significantly different between established and stable and the unstable lines (Fig. S3, Table S3, p < 0.0001).

In the first experimental year, the average meiotic pairing behaviour of the putatively stable resynthesized lines was 0.41 I + 17.10 II + 0.66 multivalents, in contrast to putatively unstable lines with 0.28 I + 11.52 II + 2.33 multivalents. Similar results were observed in the second year in the following generation of progeny, with 0.25 I + 17.81 II + 0.36 multivalents across the putatively stable lines and 1.07 I + 10.78 II + 2.48 multivalents in the putatively unstable resynthesized lines. As we hypothesized, the putatively stable resynthesized lines behaved similarly to established *B. napus* “Drakkar”, where we observed 0.26 I + 17.30 II + 0.52 multivalents (Table S3).

### “Intermediate" lines with 1–3 CNVs fell into two distinct meiotic behaviour classes (stable and unstable)

Five lines, which were classed as intermediate based on the presence of 1–3 novel CNVs, exhibited either “stable” or “unstable” phenotypic behaviour in terms of chromosome pairing. Out of the five intermediate lines, two (R8 and H327) showed normal bivalent formation (86% and 93% respectively), similar to the putatively stable lines. By contrast, the other three lines (R140, J122 and H4) exhibited lower bivalent frequencies (54%, 63% and 66% respectively), similar to the unstable lines. Based on present phenotypic results, we re-categorized these intermediate lines accordingly as putatively stable and unstable lines (Fig. 2).

### Genotype-specific differences between lines within the “stable” group and within the “unstable” group for chromosome pairing behaviour at diakinesis

We observed statistically significant differences of chromosome pairing behaviour between some of the putatively stable resynthesized lines (Fig. S4, S6) as well as between some of the putatively unstable resynthesized lines (Fig. S5, S7). Specifically, H327 (originally identified as intermediate) showed significantly lower bivalent frequencies than stable lines J154 and R8 in the first generation, although the other lines were not significantly different from each other (Fig. S4). Significant differences between multiple stable lines were also observed for multivalent and univalent frequencies (Fig. S4). However, in the next generation, there were no significant differences observed between putatively stable resynthesized lines for bivalent or multivalent frequencies, and only L16 had significantly higher univalent frequencies (Fig. S6). For the putatively unstable resynthesized lines, only OLY21 was statistically significantly different to the other four lines for bivalent frequencies in the first generation (Fig. S5) and K241 in the second generation (Fig. S7), although there were more differences in both generations for multivalent and univalent frequencies (Fig. S5, S7).

### Meiotic progression in stable resynthesized lines is similar to that in established *B. napus*, but contrasts with that of putatively unstable resynthesized lines

**Fig. 3:**
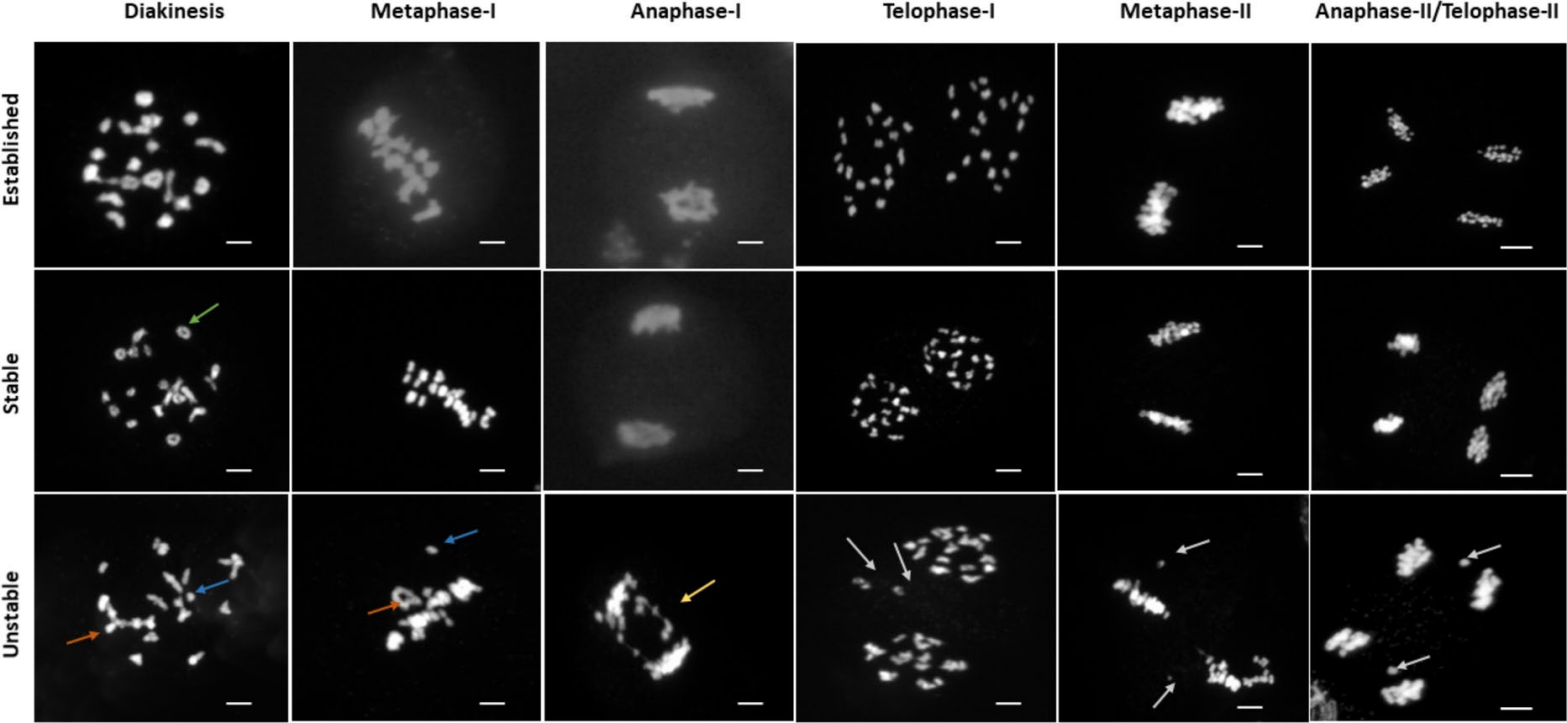
Meiotic progression in established, putatively stable and putatively unstable resynthesized *Brassica napus.* Green arrows: bivalent, blue arrows: univalent, orange arrow: multivalent, yellow arrows: anaphase bridge, white arrows: chromosome laggards. Bar = 10 µm

We looked into the different stages of meiosis I and meiosis II to understand the meiotic progression in different putatively stable and unstable lines of resynthesized rapeseed and in established rapeseed. Here, we observed relatively normal and regular meiosis in putatively stable line “L16”, which was quite similar with that of established *B. napus* “Drakkar”, but differed with that of putatively unstable resynthesized line “OLY21” (Fig. 3). Specifically, the unstable line showed higher frequencies of multivalents at diakinesis, as previously mentioned (Fig. 2, Table S3), and these multivalents were not resolved but were carried forward to metaphase I. Subsequently at anaphase I, we observed another type of structural chromosome aberration in the unstable line, namely “anaphase bridges”, which can result from crossovers between non-homologous chromosomes where two centromeres end up on the same chromatid. As well, unequal segregation ratios of chromosomes were observed at telophase I in the unstable line, indicating failure to segregate chromosomes correctly to different poles. Chromosome laggards were also observed at metaphase II and anaphase II/telophase II of meiosis II (Fig. 3), which can represent acentric fragments following non-homologous crossovers or univalent chromosomes which do not correctly segregate to the poles.

### Non-homologous multivalents involving chromosomes A1 and C1 were common but much more frequent in unstable resynthesized lines compared to established and stable lines

**Fig. 4:**
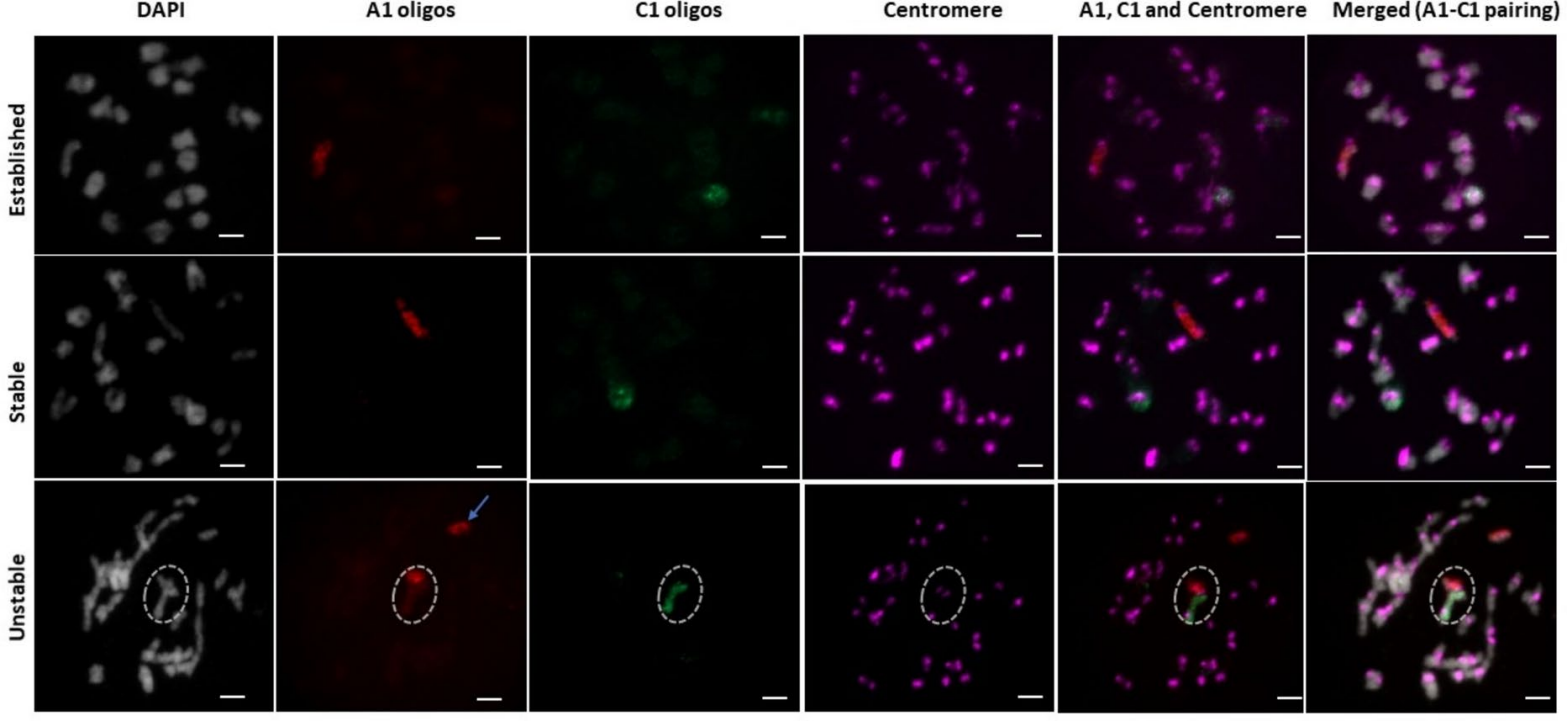
A1-C1 chromosome pairing associations at diakinesis in established, putatively stable and unstable resynthesized *Brassica napus* lines. A1: red signal, C1: green signal, centromere: purple signal, blue arrow: univalent, dotted circle: A1-C1 trivalent (A1: univalent, C1: bivalent). Bar = 10 µm

Homoeologous pairing is one of the most probable causes for meiotic instability, therefore we analysed more specifically associations between chromosome pairs A1 and C1, which have the highest known homoeology in the *B. napus* genome (Parkin et al. 2003). We applied oligo painting probes specific to the entire chromosome lengths of A1 and C1 as well as centromere-specific probes in order to analyse the frequency of A1-C1 associations in diakinesis. The average frequency of A1—C1 pairing was approximately 13% in established *B. napus* “Drakkar”, not significantly different from the approximately 18% inferred A1-C1 pairing in putatively stable resynthesized line “L16”, but contrasting significantly with the 46% frequency observed in putatively unstable line “OLY21” (p < 0.05, Fisher’s exact test for count data; Table S4 and Fig. 4).

### Correlations between meiotic behaviour and seed fertility

**Fig. 5:**
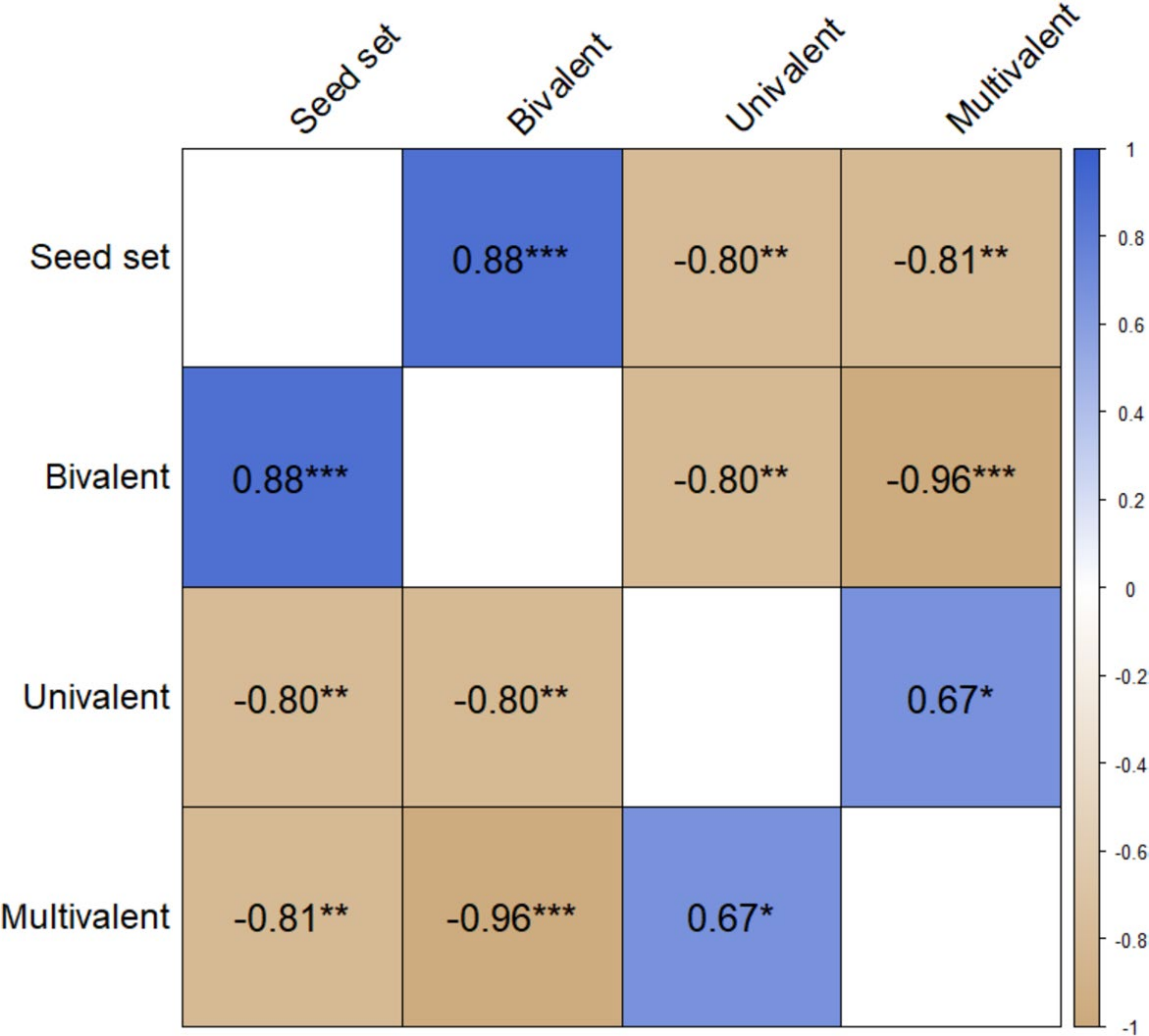
Correlations between different meiotic configuration and seed fertility in resynthesized *B. napus* (Pearson correlation, * p < 0.05, ** p < 0.01, *** p < 0.001)

Across all lines assessed, seed fertility was significantly positively correlated with bivalent formation (r = 0.88, p = 0.00066), and significantly negatively correlated with both univalent formation (r = −0.80, p = 0.0058) and multivalent formation (r = −0.81, p = 0.0048). Correlations within each of the “stable” and “unstable” groups (five lines each) were not significant (Fig. S8; Fig. S9). Unsurprisingly, meiotic configuration frequencies were also strongly significantly correlated across all lines: multivalents and bivalents were correlated at r = −0.96 (p < 0.0001), bivalents and univalents at r = −0.80 (p = 0.0053), and univalents and multivalents at r = 0.67 (p = 0.034) (Fig. 5).

## Discussion

Here, we provide the first confirmation of stable meiosis in a subset of resynthesized rapeseed lines (*B. rapa* × *B. oleracea*), comparable to that of established *B. napus*. By contrast, unstable resynthesized rapeseed lines were characterized by high frequencies of multivalents: pairing between highly similar homoeologous chromosomes A1 and C1 occurred in half of all PMCs, suggesting most multivalent formation could be attributed to pairing between homoeologous chromosomes. Additional aberrations such as acentric chromosome fragments, anaphase I chromosome bridges and unequal chromosome segregation in the unstable resynthesized lines then occurred putatively as a consequence of these non-homologous multivalents, and lower fertility was also observed in our unstable resynthesized lines compared to our stable lines. All individual resynthesized lines could be qualitatively categorised into “stable” and “unstable” on the basis of diakinesis chromosome pairing behaviour. This qualitative (stable/unstable) rather than quantitative (more or less stable on a spectrum) classification suggests a single major gene effect might differentiate these two categories, potentially supporting previous studies suggesting that one locus might be predominantly responsible for the meiotic stability trait in *B. napus* (Jenczewski et al. 2003; Higgins et al. 2021). However, we also detected some minor but significant meiotic differences between genotypes within each of the stable and unstable groups (so between stable genotypes or between unstable genotypes), which may suggest a more complex genetic background to this trait, as also observed by Katche et al., (2023a, 2023b) based on frequencies of non-homologous recombination outcomes (CNVs), and as supported by previous QTL mapping results (Liu et al. 2006; Higgins et al. 2021). Similar results were reported by Heneen et al., (1995), who observed differences in bivalent and multivalent frequencies in four different resynthesized *B. napus* lines depending upon the genetic background of the parental species, and by Katche et al. (2023b) who found differences in putative meiotic stability between early-generation synthetic lines with different combinations of shared *B. rapa* and *B. oleracea* parents. Our results provide an excellent basis to further investigate the genetic mechanisms underlying meiotic stability in allopolyploid *Brassica napus*, putatively by production of biparental mapping populations between stable and unstable resynthesized lines and/or by validation of specific allelic variants of meiosis genes or gene expression differences between stable and unstable genotypes.

Unstable resynthesized lines were characterized by high frequencies of multivalents in comparison to stable and established *B. napus*, but not by increased univalent formation. Stable resynthesized lines and established *B. napus* Drakkar showed similar frequencies of bivalents (both 91%), univalents (2% and 1%) and multivalents (3% in both) at diakinesis, in line with reports of other studies in established cultivars of *B. napus* (Jenczewski et al. 2003; Udall et al. 2005; Sheidai et al. 2006). We observed no significant difference between stable and unstable lines for univalent frequency in the first generation. Minor differences in univalent frequencies were observed in the second generation, but were putatively attributable to aneuploidy (presence of chromosomes without homologous pairing partners). Similarly, Xiong et al., (2021) observed low frequencies of univalents in resynthesized rapeseed lines. However, we observed highly significant differences between stable and unstable lines for multivalent frequency in both generations, with > 2 multivalents per PMC on average in unstable resynthesized lines. Our results suggest that reduction of multivalent formation plays a major role in meiotic and genomic instability in *Brassica* polyploids.

We assessed progression through meiotic stages of meiosis I and meiosis II such as diakinesis, metaphase, anaphase and/or telophase in order to determine if stage-specific failures to complete normal and regular meiosis and develop functional gametocytes were present. Our detailed comparative meiotic progression study between putatively stable (L16) and unstable (OLY21) resynthesized lines identified regular meiotic progression in the stable resynthesized line, similar to established cultivar “Drakkar”. By contrast, higher frequencies of multivalents at diakinesis and misalignment of bivalents at metaphase I were observed in the unstable resynthesized line, suggesting that homologous and homoeologous chromosomes were unable to sort, synapse and properly recombine in the early stages of prophase. Similarly, (Grandont et al. 2014) reported early and effective sorting of homologous and homoeologous chromosomes in early prophase in two different *B. napus* accessions (although these accessions showed different frequencies of homoeologous A-C recombination as allohaploids). Subsequently, meiotic irregularities resulting from early meiosis carry forward through meiosis I and meiosis II. In the unstable line, multivalents which are unable to resolve putatively continue to anaphase I and/or telophase I in the form of anaphase bridges, where the chromosomes are unable to segregate equally to opposite poles, along with chromosome fragments and laggards which putatively result from univalents. These univalents/chromosome laggards carry forward to metaphase II and anaphase II and/or telophase II. By contrast, the stable line showed relatively normal progression through anaphase I/telophase I to anaphase II/telophase II without chromosomal abnormalities, indicating that the initial prophase I step is essential to maintain stable and regular meiosis, as has also been previously proposed (Grandont et al. 2014). Studies in ACC hybrids, which also have frequent multivalent formation as well as high numbers of univalents from the haploid genome, also show similar meiotic issues: Yang et al., (2017) reported partially tangled chromosomes at diakinesis, jumbled alignment at metaphase I with frequent chromosome bridges, unequal segregation and laggards in anaphase I and anaphase II, in contrast to parental lines diploid *B. oleracea* and allotetraploid *B. napus*, where the chromosomes were paired as bivalents, orderly aligned at the equatorial plate in metaphase and equally segregated.

While statistically similar frequencies of A1/C1 homoeologous associations were observed in stable resynthesized lines (18%) and established *B. napus* (13%), nearly half (46%) of PMCs in the unstable resynthesized line showed A1/C1 associations. Homoeologous chromosomes A1 and C1 share collinearity and homology along the length of the chromosome, which leads to higher chances of homoeologous recombination than between other, more rearranged A/C genome homoeologous (Higgins et al. 2021; Udall et al. 2005). Analysis of these A1/C1 associations therefore allowed us to estimate homoeologous pairing and make general assumptions. High frequencies of homoeologous recombination between the A and C genomes have also been observed in other studies of resynthesized rapeseed (Parkin et al. 1995; Sharpe et al. 1995; Udall et al. 2005), supporting our results. Xiong et al., (2021) reported 49—57% A1/C1 homoeologous pairing in resynthesized *B. napus* in both the S1 and S11 generations, very similar to our current observations.

We observed relatively high frequencies of A1/C1 homoeologous associations in the putatively stable resynthesized line and established cultivar, even though these frequencies were lower than in the unstable resynthesized lines. Frequent homoeologous translocations in established *B. napus* were reported as early as 1995 (Parkin et al. 1995; Sharpe et al. 1995), although at lower rates than in synthetic *B. napus,* suggesting that *B. napus*, as a relatively young allopolyploid, is not fully able to prevent homoeologous pairing between the A and C genomes (Jenczewski & Alix 2004; Sourdille & Jenczewski 2021). Although pre-existing homoeologous recombination events in either Drakkar or in our putatively stable resynthesized lines could cause frequent multivalents. Large-scale (> 1 Mb) translocations were not visible from either our chromosome oligo painting method or previous SNP array data results (Katche et al. 2023a) for these lines.

Overall, our results indicate that the putatively stable resynthesized line prefer homologous recombination over homoeologous recombination similarly to established *B. napus*. Although we only assessed one line of each of the stable, unstable and established types for A1/C1 homoeologous recombination, such that we cannot completely rule out genotype-specific factors, it is highly likely that this finding of homoeologous pairing reduction could be a general event at least to the set of stable synthetic lines we investigated in the present study. Previous studies have also suggested that A/C pairing occurs between all homoeologous chromosome regions genome-wide in direct relation to degree of homoeology in a mostly genotype-independent fashion (Nicolas et al. 2007, 2009, 2012; Mason et al. 2014), supporting a general mechanism acting to prevent homoeologous recombination in established but not novel resynthesized lines.

All our stable lines were later generation lines and our unstable lines were all early generation, such that further investigation of generational effects on stability may also be warranted. It is unclear from previous studies of resynthesized and synthetic *Brassica* if meiotic stability can improve generationally when starting from homozygous lines, where no segregation for allelic variation is possible. In studies where later-generation, putatively more homozygous lines show better meiotic stability than earlier generation lines (Tian et al. 2010 in *B. rapa* by *B. carinata* allohexaploids, as well as Katche et al. 2023a), selection putatively occurs in the early generations, such that unstable lines fail to produce seeds and are not able to be propagated to later generations, while lines which are initially more stable produce more seeds and survive to later generations. Xiong et al., (2021) also observed no significant difference in non-homologous chromosome pairing frequency over 11 generations in homozygous resynthesized *B. napus* (20% in the S1 and 16% in the S11 generation). Nevertheless, substantial changes are also possible in synthetic hybrids as a result of “genome shock” resulting from the hybridization event, such as transcriptional regulation changes (Gaeta et al. 2007), chromosome rearrangements (Szadkowski et al. 2010), activation of transposable elements (Zou et al. 2011) and proteomic changes (Albertin et al. 2006), all of which may create novel genetic variation which may be acted on by selection from early to late generation lines and provide a mechanism for stabilisation of meiosis. Limitations of small sample sizes and diverse genetic backgrounds in the current study suggest further investigation of generational effects on stability within specific lineages would be necessary to confirm this hypothesis.

We observed no major differences between the two generations we grew in our study, but we did find that in the unstable lines, univalent frequency in the second experimental year was significantly higher than in the first experimental year generation. Szadkowski et al., (2010) also observed high frequencies of univalents in S1 resynthesized *B. napus*, where this was attributed to aneuploidy (absence of a homologous pairing partner is expected to result in a univalent or trivalent) although Xiong et al., (2021) did not observe many univalents in their synthetic *B. napus* lines. Mwathi et al., (2019) also found that meiotically unstable homozygous *Brassica* allohexaploids showed increased frequencies of chromosome rearrangements and reduced fertility from one generation to the next, and hypothesised that initial chromosome rearrangements also resulted in more rearrangements. Similar effects may be responsible for the increased meiotic instability observed from one generation to the next in our unstable resynthesized lines.

## Supplementary Information

Below is the link to the electronic supplementary material.

Supplementary file1 (PDF 716 KB)

Supplementary file2 (XLSX 68 KB)

## Data Availability

All data generated by this project is available in the manuscript and supplementary material.
