# Supplementary material for: Stable resynthesized Brassica napus lines show similar meiotic behaviour to established B. napus
Source: Chromosome Res. 2026 Apr 7;34(1):8. doi: 10.1007/s10577-026-09799-1 (PMC13056725; doi:10.1007/s10577-026-09799-1)
Supplement: Supplementary file 1 — Supplementary file1 (PDF 716 KB) [file 10577_2026_9799_MOESM1_ESM.pdf]

Supplementary Figures

"Stable resynthesized *Brassica napus* lines show similar meiotic behaviour to established *B. napus*"

Vinita Ramtekey, Elizabeth Ihien Katche, Mariana Baez, Zhenling Lv and Annaliese S. Mason

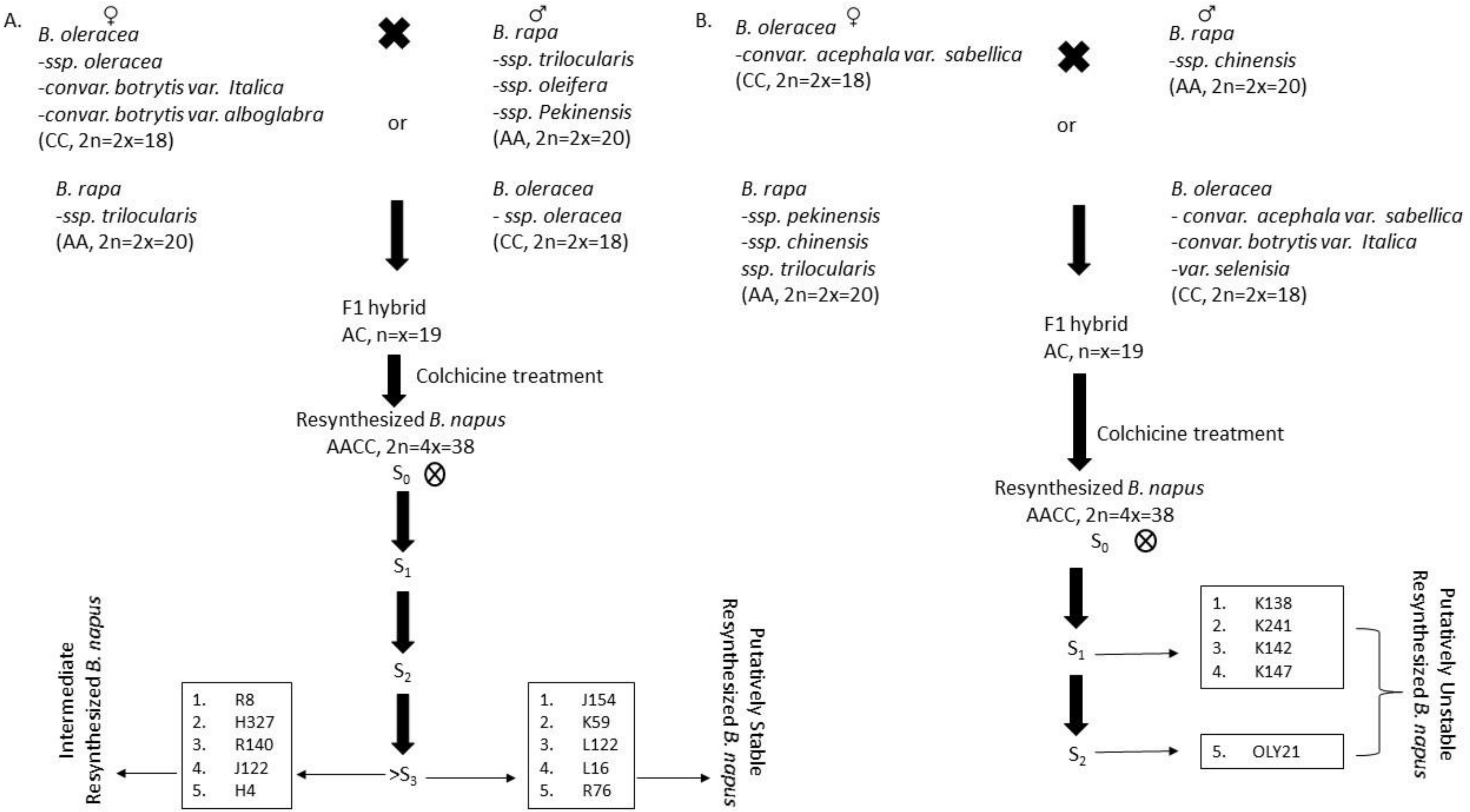

Fig. S1: Pedigree of putatively stable, intermediate and unstable resynthesized *B. napus*. Selected putatively stable, intermediate and unstable lines are produced by hand pollination followed by embryo rescue between *B. rapa* and *B. oleracea* cultivars followed by colchicine treatment then one or more round of self-pollination. A. Stable and intermediate lines belong to later generation (>S3); and B. Unstable belong to early generation (S1 and S2).

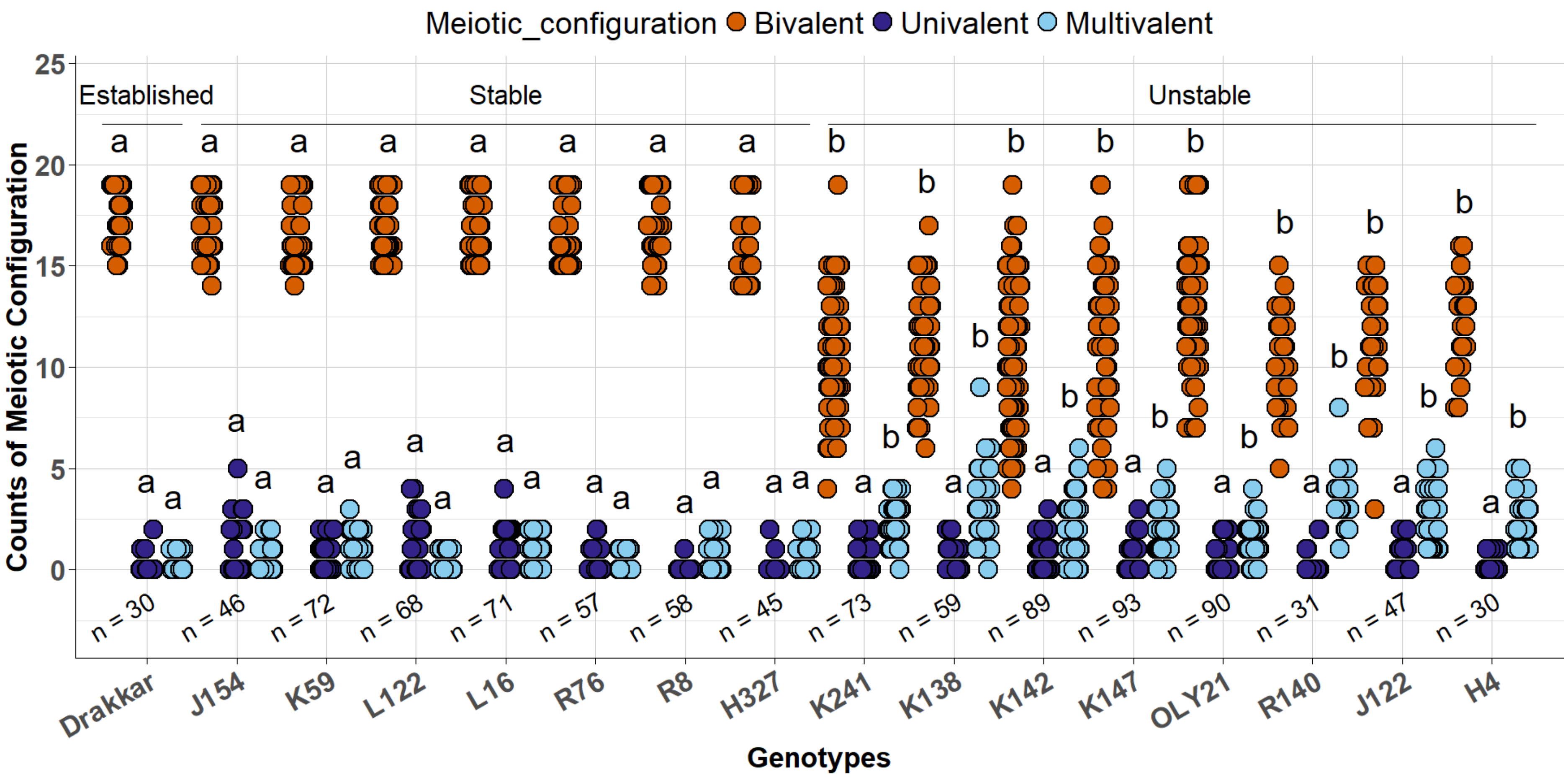

Fig. S2: Meiotic chromosome pairing behaviour in established *Brassica napus* cultivar "Drakkar" and in putatively stable and unstable resynthesized *B. napus* lines (2022-23). Letters indicate significant differences between lines for each of bivalent, univalent and multivalent frequencies (Kruskal-Wallis test followed by Dunn's post hoc test, $p < 0.05$). n = number of meiotic cells. Each dot represents a single meiotic cell.

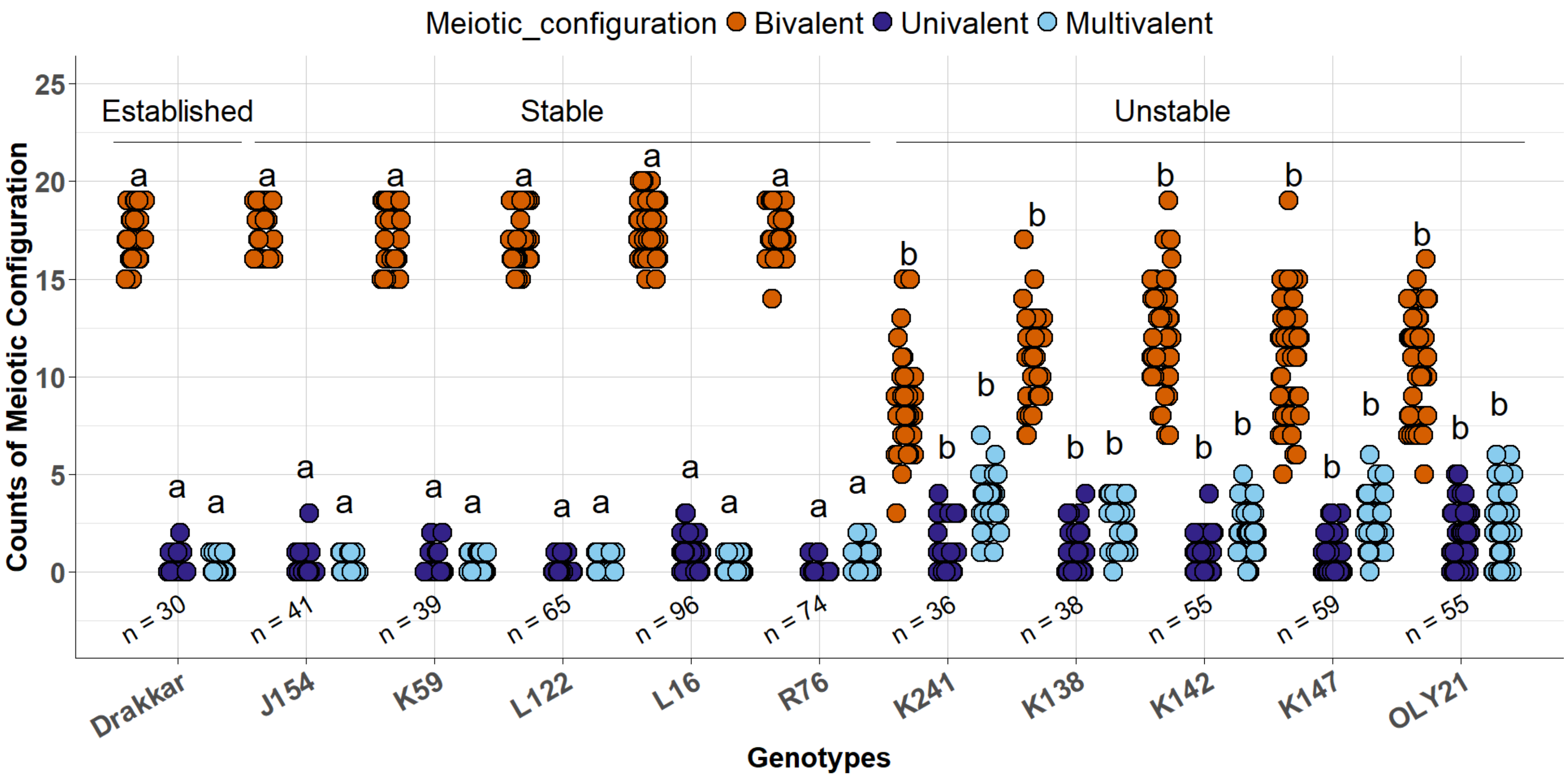

Fig. S3: Meiotic chromosome pairing behaviour in established *Brassica napus* cultivar "Drakkar" and in putatively stable and unstable resynthesized *B. napus* lines (2023-24). Letters indicate significant differences between lines for each of bivalent, univalent and multivalent frequencies (Kruskal-Wallis test followed by Dunn's post hoc test, $p < 0.05$). n = number of meiotic cells. Each dot represents a single meiotic cell.

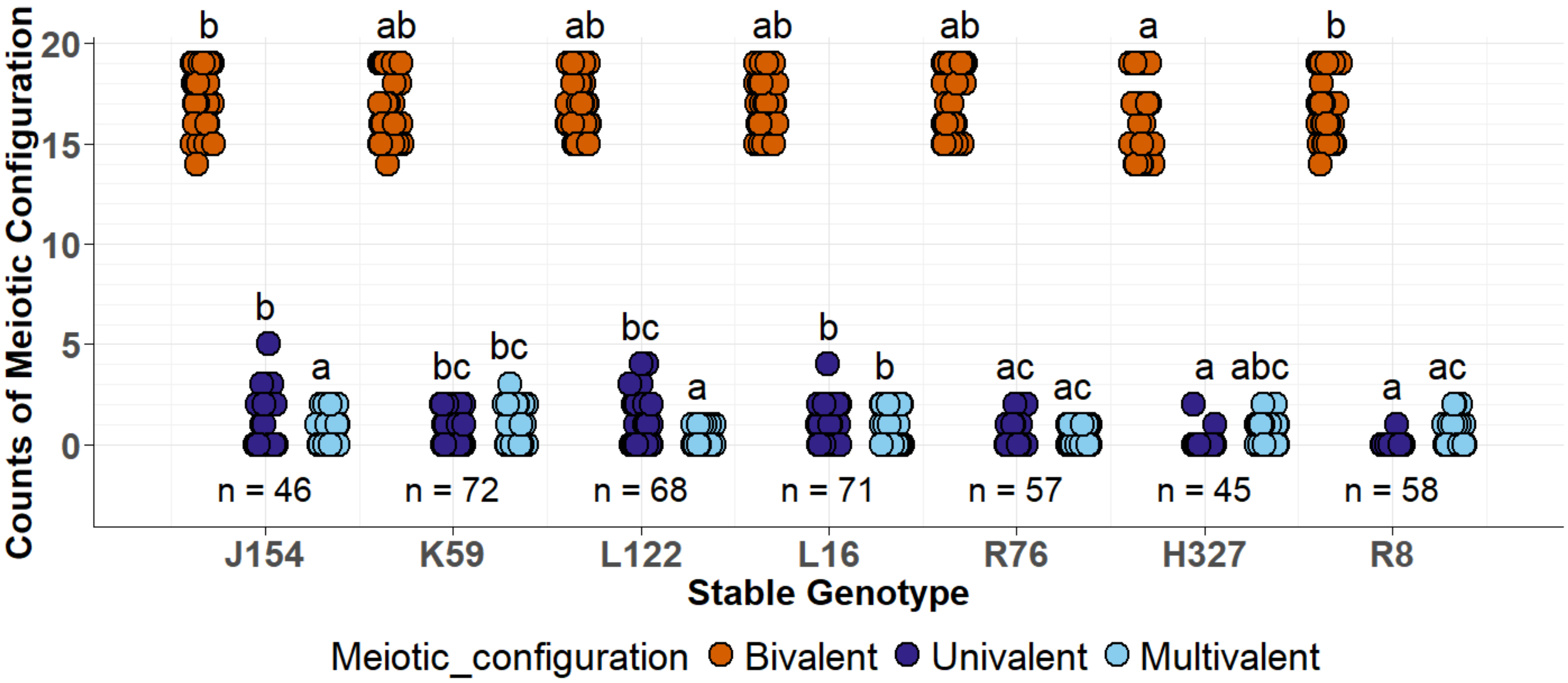

Fig. S4: Meiotic chromosome pairing behaviour in putatively stable resynthesized *B. napus* lines (2022-23). Letters indicate significant differences between lines for each of bivalent, univalent and multivalent frequencies (Kruskal-Wallis test followed by Dunn's post hoc test, $p < 0.05$). n = number of meiotic cells. Each dot represents a single meiotic cell.

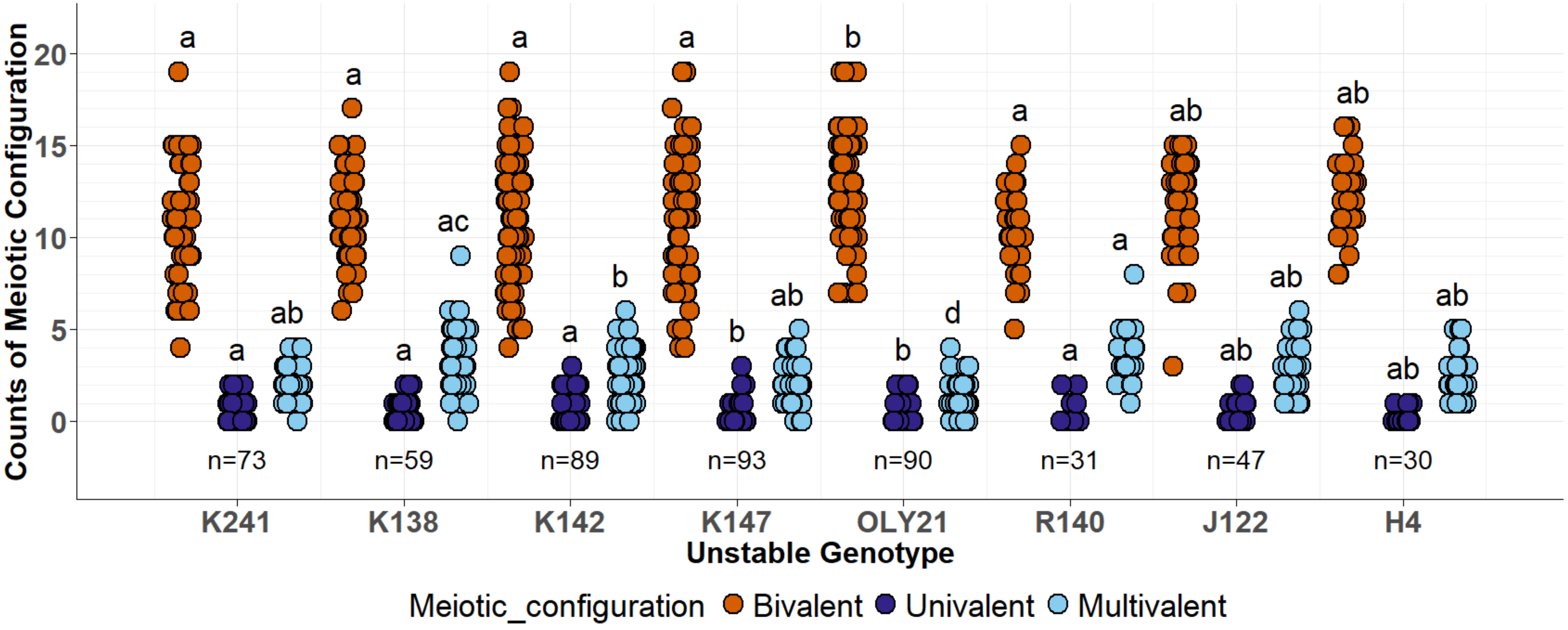

Fig. S5: Meiotic chromosome pairing behaviour in putatively unstable resynthesized *B. napus* lines (2022-23). Letters indicate significant differences between lines for each of bivalent, univalent and multivalent frequencies (Kruskal-Wallis test followed by Dunn's post hoc test, $p < 0.05$). n = number of meiotic cells. Each dot represents a single meiotic cell.

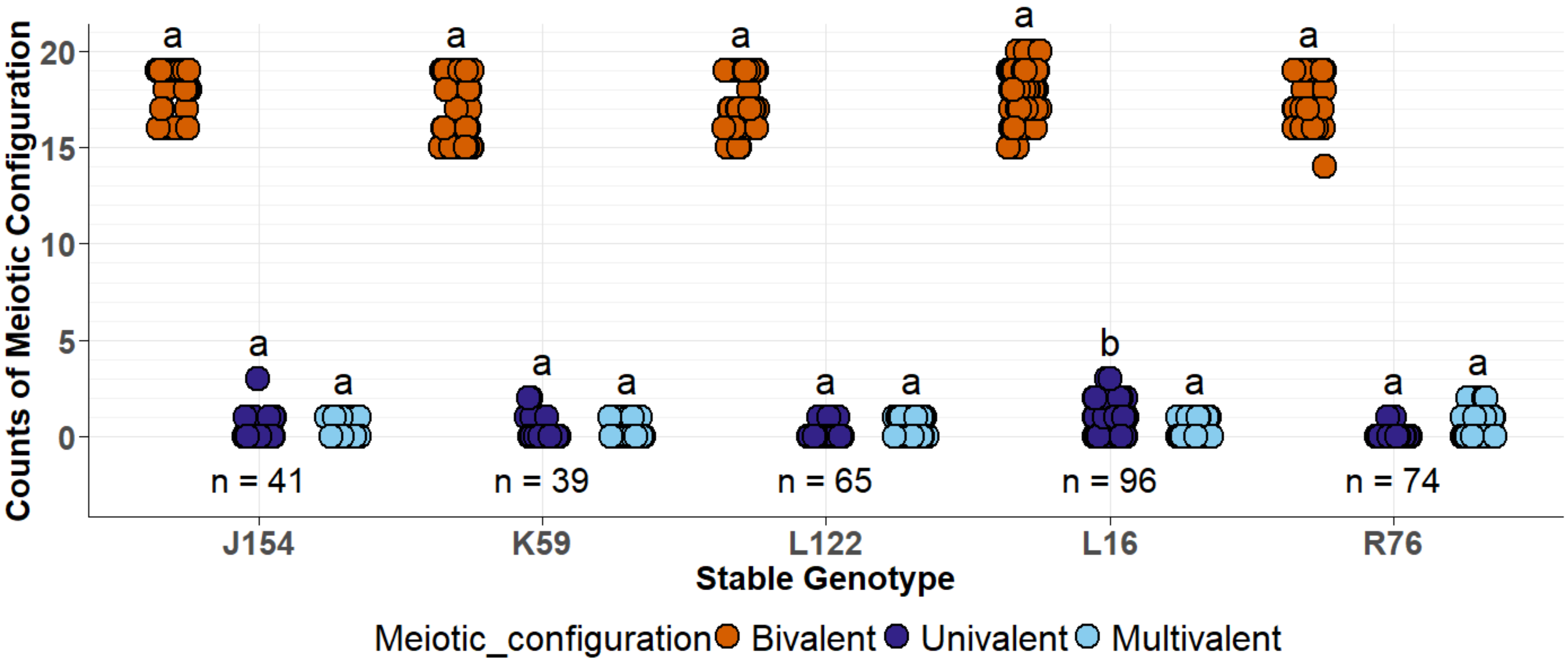

Fig. S6: Meiotic chromosome pairing behaviour in putatively stable resynthesized *B. napus* lines (2023-24). Letters indicate significant differences between lines for each of bivalent, univalent and multivalent frequencies (Kruskal-Wallis test followed by Dunn's post hoc test, $p < 0.05$). n = number of meiotic cells. Each dot represents a single meiotic cell.

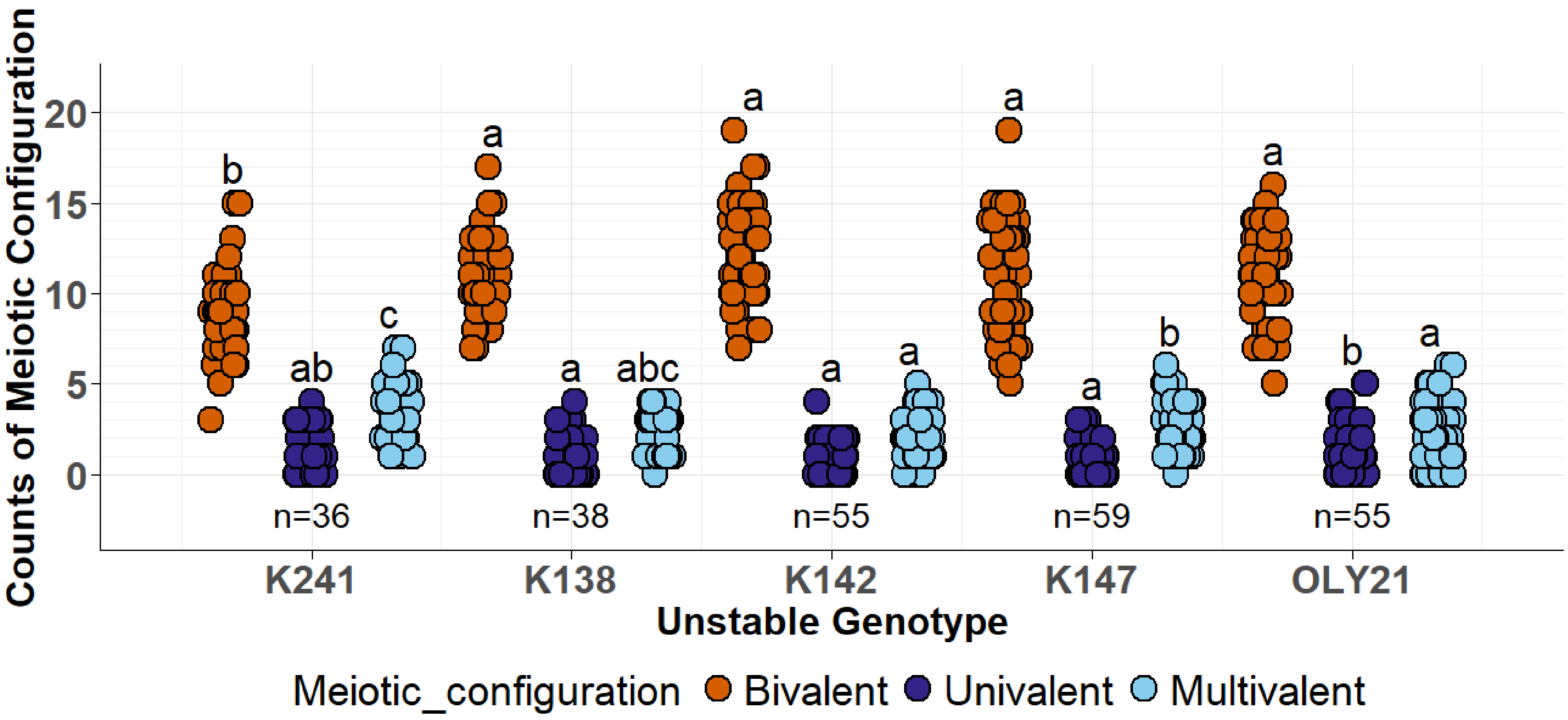

Fig. S7: Meiotic chromosome pairing behaviour in putatively unstable resynthesized *B. napus* lines (2023-24). Letters indicate significant differences between lines for each of bivalent, univalent and multivalent frequencies (Kruskal-Wallis test followed by Dunn's post hoc test, $p < 0.05$). n = number of meiotic cells. Each dot represents a single meiotic cell.

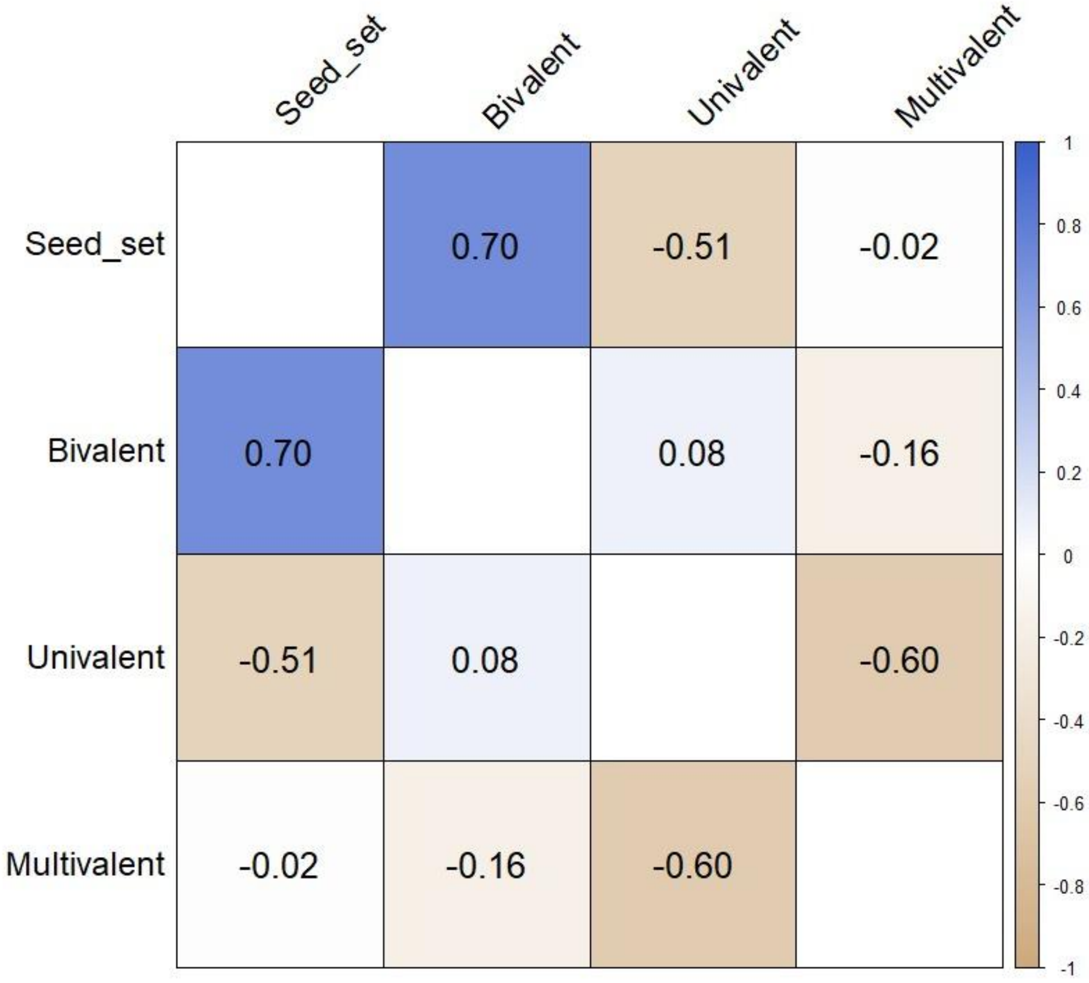

Fig. S8: Correlation between different meiotic configuration and seed fertility in putatively stable resynthesized *B. napus* (Pearson correlation, P<0.05).

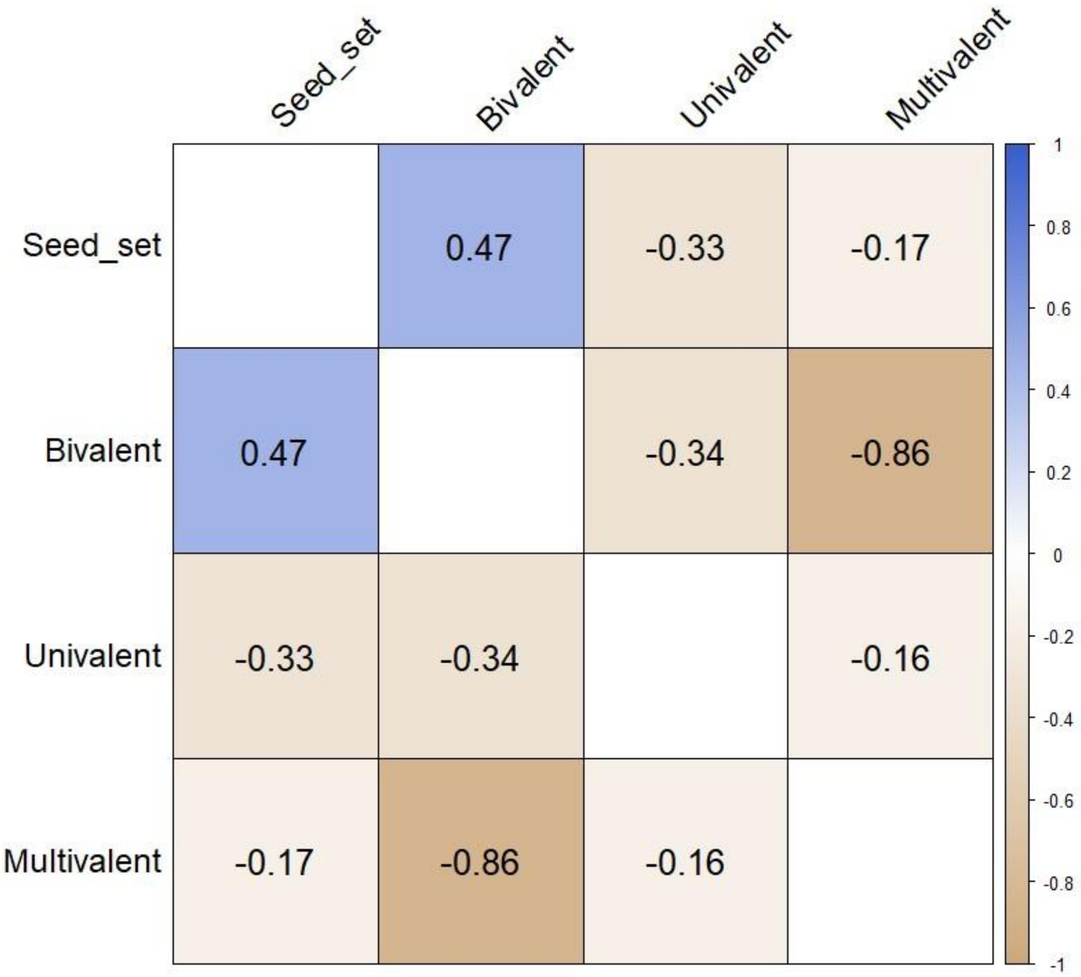

Fig. S9: Correlation between different meiotic configuration and seed fertility in putatively unstable resynthesized *B. napus* (Pearson correlation, P<0.05).
